# Application of liquid biopsy for prognostic status of cancers and drug response

**DOI:** 10.20517/cdr.2020.96

**Published:** 2020-11-03

**Authors:** Dario Marchetti

**Affiliations:** Division of Molecular Medicine, Department of Internal Medicine, Comprehensive Cancer Center, The University of New Mexico Health Sciences Center, Albuquerque, NM 87131, USA.

The vast majority of cancer deaths (> 94%) are due to the development of metastatic disease with a considerable fraction of patients experiencing metastatic relapse within five years from primary tumor resection, despite not having any detectable metastasis at that time. Circulating tumor cells (CTCs) are shed from primary/metastatic tumors, circulate in the peripheral vasculature, disseminate to distant tissues, and extravasate by adapting to the new organ microenvironment, followed by tissue colonization generating metastasis. However, the complexities of the metastatic cascade in cancer patients and the elucidation of blood-based mechanisms underlying metastatic onset remain critical challenges. Because CTC dissemination mostly occurs through the blood, and only a minute proportion of CTCs can generate metastasis (metastasis-competent CTCs), investigating the biology of CTCs for biomarker discovery is critically relevant, which is one of the most promising areas of modern oncology research. Equally - beyond CTCs - there have also been many advances to detecting and interrogating blood-based, tumor-specific biomarkers such as circulating tumor DNA, circulating exosomes and extracellular vesicles, tumor-educated platelets, circulating cell-free microRNA, proteins, and metabolites. The use of these analytes to obtain valuable diagnostic information from blood/body fluids rather than from a tissue biopsy sample is broadly referred as “liquid biopsy”. The development and application of “liquid biopsy” testing in cancer patients is a novel and powerful diagnostic concept to monitor cancer progression directly, in real time, and non-invasively. However, many scientific, procedural, and technical limitations must be overcome before the promise of “liquid biopsy” can be fulfilled and successfully and consistently implemented in the clinics. The objective of this special issue, “Application of Liquid Biopsy for Prognostic Status of Cancers and Drug Response”, is to publish the latest findings of liquid biopsy research and clinical application as prognostic indicators of therapy response. The contributions outlining discoveries in this area and the development/application of liquid biopsy testing either in pre-clinical or clinical models will include reviews and commentaries updating on the relevance of “liquid biopsy” and analytes validity in the prevention or treatment of different tumor types. This Special Issue will also include research articles presenting innovative advances impacting the field on all aspects and components of liquid biopsy. All manuscript submissions will be given a fair evaluation by undergoing rigorous peer-review, and they will be published if considered acceptable for scientific divulgation.

## Declarations

### Copyright

© The Author(s) 2020.

